# Functional roles of Phe^12^ of deacetyl-thymosin β4 in the impaired blastogenic response of uraemic T-lymphocytes

**DOI:** 10.1080/09629359791956

**Published:** 1997-02

**Authors:** T. Abiko, H. Sekino

**Affiliations:** 1 Kidney Research Laboratory Kojinkai 1– 6 Tsutsujigaoka 2-chome Miyagino-ku Sendai 980 Japan

## Abstract

Phe^12^ of deacetyl-thymosin β_4_ is one of the structural essentials for restorative effect on the impaired blastogenic response of uraemic T-lymphocytes. In order to evaluate the functional roles of this phenyl group in the restorative effect on impaired T-lymphocytes, two analogues, [1- Nal^12^]deacetyl-thymosin β_4_ and [Cha^12^]deacetyl4 thymosin β_4_, were synthesized by a solid-phase method and evaluated for restorative effect on the impaired blastogenic response of uraemic T-lymphocytes. The results indicated that [1-Nal^12^]deacetyl-thymosin β_4_ which had a bulky naphthyl ring showed a stronger restorative effect than that of deacetyl-thymosin β_4_, but it was slightly weaker than that of [Phe(4F)^12^]deacetyl-thymosin β_4_. However, [Cha^12^]deacetyl-thymosin β_4_ showed no restorative effect on the impaired blastogenic response of uraemic T-lymphocytes.


Mediators of Inflammation, 6, 64–68 (1997)

### Functional roles of $Phe^{12}$ of deacetyl-thymosin $\beta_4$ in the impaired blastogenic response of uraemic T-lymphocytes

T. Abiko[CA] and H. Sekino

Kidney Research Laboratory, Kojinkai, 1–6 Tsutsujigaoka 2-chome, Miyagino-ku, Sendai 980, Japan

[CA]Corresponding Author
Fax: (+81) 22 2972414

**PHE$^{12}$ of deacetyl-thymosin $\beta_4$ is one of the structural essentials for restorative effect on the impaired blastogenic response of uraemic T-lymphocytes. In order to evaluate the functional roles of this phenyl group in the restorative effect on impaired T-lymphocytes, two analogues, [1-Nal$^{12}$]deacetyl-thymosin $\beta_4$ and [Cha$^{12}$]deacetyl-thymosin $\beta_4$, were synthesized by a solid-phase method and evaluated for restorative effect on the impaired blastogenic response of uraemic T-lymphocytes. The results indicated that [1-Nal$^{12}$]deacetyl-thymosin $\beta_4$ which had a bulky naphthyl ring showed a stronger restorative effect than that of deacetyl-thymosin $\beta_4$, but it was slightly weaker than that of [Phe(4F)$^{12}$]deacetyl-thymosin $\beta_4$. However, [Cha$^{12}$]deacetyl-thymosin $\beta_4$ showed no restorative effect on the impaired blastogenic response of uraemic T-lymphocytes.**

**Key words:** Blastogenic response, Deacetyl-thymosin $\beta_4$ analogue synthesis, Impaired T-lymphocyte, Restorative effect, Uraemic patient

### Abbreviations

Boc, tert-butyloxycarbonyl; tBu, tert-butyl; DMF, *N,N*-dimethylformamide; TFA, trifluoroacetic acid; Fmoc, 9-fluorenylmethoxycarbonyl; HOBT, 1-hydroxybenzotriazole; DCC, dicyclohexylcarbodiimide; $HBF_4$, tetrafluoroboric acid; EDT, ethane-1,2-dithiol; AcOH, acetic acid; HPLC, high-performance liquid chromatography; TLC, thin-layer chromatography; 1-Nal, 1-naphthylalanine; Cha, cyclohexylalanine; PHA, phytohaemagglutinin; RPMI, Rosewell Park Memorial Institute; SDS, sodium dodecyl sulphate; PBS, phosphate-buffered saline; FCS, fetal calf serum; FAB-MS, fast atom bombardment mass spectrometry; Pam, phenylacetoamido-methyl; Ac, acetyl.

### Introduction

The impairment of immunological responsiveness in uraemic patients is well known. All aspects of the immune response appear to be affected by the uraemic state. The numbers, subpopulations and reactivities of circulating lymphocytes may be altered by uraemia.[1,2] This impairment has been implicated in easy susceptibility to infections and increased incidence of malignancy.

Thymosin $\beta_4$, an N-terminal acetylated peptide containing 43 amino acid residues, was first isolated from calf thymus by Low *et al.*[3] and has the following amino acid sequence: Ac-Ser-Asp-Lys-Pro-Asp-Met-Ala-Glu-Ile-Glu-Lys-Phe-Asp-Lys-Ser-Lys-Lys-Thr-Glu-Thr-Gln-Glu-Lys-Asn-Pro-Leu-Pro-Ser-Lys-Leu-Lys-Glu-Thr-Ile-Glu-Gln-Glu-Lys-Gln-Ala-Gly-Glu-Ser-OH. This peptide exhibits several biological activities that are important for maturation and functioning of the immune systems.[4]

Previously[5–7] we reported syntheses of deacetyl-thymosin $\beta_4$ and its fragments and that some of the fragments could have a restorative effect on the impaired cell-mediated immunological functions. We also noticed that the acetyl group at the N-terminal serine residue of thymosin $\beta_4$, is not required for the restorative effect on the impaired cell-mediated immunological functions.[5]

In an earlier paper,[8] we reported that the synthetic [Phe(4F)$^{12}$]deacetyl-thymosin $\beta_4$ exhibited stronger restorative effect on the impaired blastogenic response of T-lymphocytes isolated from uraemic patients than that of our synthetic deacetyl-thymosin $\beta_4$. In this study, the strong electron-withdrawing fluoride atom on the para-position of the aromatic ring results in an analogue that possesses stronger activity than that of the parent molecule.[8] This result seems to suggest that modification of the Phe residue of thymosin $\beta_4$ could produce more potent analogues capable of a restorative effect

© 1997 Rapid Science Publishers on impaired blastogenic response of T-lymphocytes.

The purpose of the present study was to synthesize two thymosin $\beta_4$ analogues, [1-Nal[12]]deacetyl-thymosin $\beta_4$ and [Cha[12]]deacetyl-thymosin $\beta_4$ by the solid-phase method and to compare the restorative effect of these two analogues on the impaired blastogenic response of uraemic T-lymphocytes.

### Materials and Methods

Fmoc-amino acid derivatives and Fmoc-Ser (tBu)-Pam-resin (0.64 mmol/g, 100–200 mesh) were purchased from Kokusan Chemical Works Ltd (Japan), Watanabe Chemical Industries Ltd (Japan), Peptide Institute Inc (Japan) and Sigma Chemical Co. (USA). TLC was effected with silica gel (Kieselgel $60F_{254}$, Merck) on precoated aluminium sheets using *n*-BuOH-AcOH-pyridine-$H_2$) = 4:1:1:2 as a solvent system. Analytical HPLC and amino acid analysis were conducted with a Shimadzu LC-6A and Hitachi 835A, respectively. The FAB-MS spectrum was obtained on a VG analytical 2AB-2SEQ spectrometer equipped with the 11-250J data system.

### Solid-phase peptide synthesis

Peptide synthesis was performed manually by the stepwise solid-phase method with a handmade peptide synthesizer, using the base-labile Fmoc group for protecting the $\alpha$-amino groups, and such acid-labile groups as the tBu for the hydroxy and carboxy groups, the Boc for the $\varepsilon$-amino groups of Lys, and the sulphoxide for Met. The peptide was assembled on Fmoc-Ser (tBu)-Pam-resin. The Emoc group was removed with 30% piperidine in DMF. Elongation of the peptide chain was carried out by the DCC-HOBT method in $CH_2Cl_2$-DMF (1:1) or in N-methyl-2-pyrrolidone. The coupling reaction and deprotection of the Fmoc group were monitored by the ninhydrin test. The general procedure for each synthetic cycle (as a starting mateial 0.64 mmol/g of Fmoc-Ser(tBu)-Pam-resin: 400 mg) was: (1) $CH_2Cl_2$ wash (twice); (2) DMF wash (twice); (3) deprotect: DMF-piperidine (7:3) for 20 min; (4) DMF wash (twice); (5) dioxane-water (2:1) wash (twice); (6) DMF wash (three times); (7) $CH_2Cl_2$ wash (three times); (8) addition of 3 eq Fmoc-amino acid, HOBT, and DCC in $CH_2Cl_2$-DMF (1:1) or N-methyl-2-pyrrolidone; (9) add 1.0 ml of diisopropylethylamine in $CH_2Cl_2$; (10) reaction for 120 min; (11) recoupled if necessary by repeating steps 7–10; (12) DMF wash (three times); (13) isopropanol wash (three times); (14) $CH_2Cl_2$ wash (four times). Whenever the ninhydrin test was still slightly positive, even after three couplings, the remaining unreacted amino groups were acetylated with $Ac_2O$-pyridine in DMF. The peptide resin (200 mg) was treated with 2 M $HBF_4$-thioanisole in TFA (7 ml) in the presence of m-cresol (218 µl, 100 eq) and EDT (524 µl, 300 eq) at 4°C for 90 min.

After the deprotection, the resin was removed by filtration and the filtrate was evaporated under reduced pressure and the residue was solidified by addition of anhydrous peroxide free ether to give a crude peptide. The resulting powder was dissolved in $H_2O$ (6 ml). The solution was treated with Amberlite CG-4B (acetate form, approximately 3 g) for 30 min, and filtered by suction and evaporated *in vacuo*. The residue was dissolved in $H_2O$ (10 ml). The solution, after addition of dithiothreitol (20 mg), was incubated at 60°C under $N_2$ gas for 36 h. The solvent was evaporated off *in vacuo* and the residue was dissolved in a small amount of 1% AcOH and then applied to a column of Sephadex G-25 (2.3 × 95 cm), which was eluted with the same solvent. Individual fractions (5 ml each) were collected and absorbancy at 230 nm was determined for each fraction. The fractions corresponding to the front main peak were combined and the solvent was removed by lyophilization. The peptide was further purified by semi-preparative PR-HPLC. The semi-preparative PR-HPLC was performed on a Nucleosil C18 column (250 × 10 mm I.D.; 7 µm particle size; Macherey Nagel). Solvent A was 0.05% TFA in water and solvent B was 60% acetonitrile in solvent A. A linear gradient was applied from 10 to 50% B during 50 min, at a flow rate of 3.0 ml/min. Detection of the peptide was set at 230 nm. The major peak was lyophilized to give the purified product. [1-Nal[12]]deacetyl-thymosin $\beta_4$; 20.3 mg (20%, calculated from the starting C-terminal amino acid). [Cha[12]]deacetyl-thymosin $\beta_4$; 22.6 mg (23%, calculated from the starting C-terminal amino acid) (Fig. 1).

### Patient selection

Three uraemic patients who needed dialysis treatment three times a week and were suffering from recurrent infectious diseases (pneumonia and tuberculosis) were selected. Examination of cellular immunocompetence of these patients revealed a significant decrease in blast-formation by PHA. $^3H$-thymidine incorporation values of these patients were 11 826, 12 042 and 12 153 cpm respectively (normal values: 41 195–42 659 cpm).

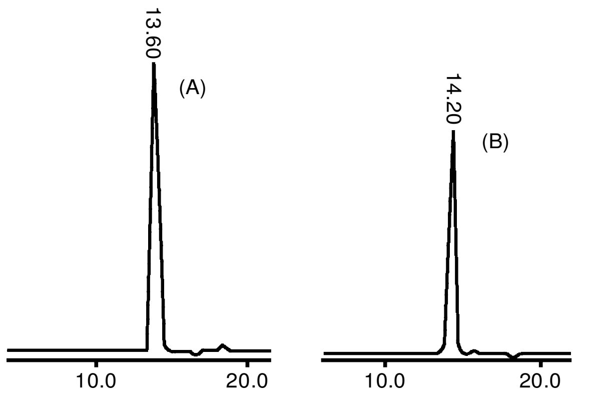

Fig. 1. HPLC profiles of (A) [1-Nal$^{12}$]deacetyl-thymosin $\beta_4$ and (B) [Cha$^{12}$]deacetyl-thymosin $\beta_4$.

Venous blood was obtained from these uraemic patients for the fluorometric blast-formation test. Venous blood samples from three healthy donors were used as a control. The fluorescence excitation spectrum was measured with an Oyo-Bunko ULOG-FLOUSPEC 11A fluorometer. Kits for the fluorometric blast-formation test were purchased from Japan Immunoresearch Laboratories Co. Ltd (Japan).

### Fluorometric blast-formation test

A 3 ml aliquot of venous blood was drawn into a syringe containing 25 U/ml of heparin and then mixed with 3 ml of PBS. Lymphocytes were isolated in a Hypaque-Ficoll gradient. Isolated lymphocytes were adjusted to $1.0 \times 10^6$/ml with PBS. The lymphocytes were cultured in 0.5 ml of RPMI 1640 (Gibco) with 10% FCS (Dainippon Pharmaceutical Co.) in microplates. Cultures of each combination were incubated at 37°C in the presence of one of the peptides in a humidified atmosphere of 5% $CO_2$ in air or 12 h and then PHA (0.125%) was added to each well and incubation was continued under the same conditions for 60 h. Lymphocytes in each well were transferred into a test tube and centrifuged for 10 min at 240 g, then the supernatant was removed. A 2 ml aliquot of 0.125% SDS was added to the residue and stirred for 20 min at room temperature; lymphocytes were completely destroyed and solubilized by this procedure. Ethidium bromide solution (2 ml) was added to the above solution and the mixture was stirred for 15 min at room temperature. The fluorescence excitation spectrum was measured as previously described.[7]

### Results

In order to construct the peptide chain, the Fmoc-based solid-phase method was employed. Fmoc-Ser(tBu)-Pam-resin was placed in the reaction vessel and the combination of piperidine treatment and DCC plus HOBT procedure served to elongate the peptide chain manually according to the usual method.

We encountered no serious difficulties during the elongation of the entire sequences of the two analogues, although the double coupling procedure was employed when the resin became positive to the ninhydrin test, after a single coupling. The coupling cycle included a capping step with acetic anhydride (5 min) to prevent the formation of deleted sequences. The amino acid compositions of the protected peptide resins thus assembled were in good agreement with those predicted by theory after acid hydrolysis with 12 N HCl-proprionic acid (1:1). The protected peptide resins thus obtained were then treated with 2 M $HBF_4$-thioanisole in TFA at 4°C for 90 min to cleave the peptide chain from the resin and at the same time to remove all side-chain protecting groups employed. The Met(O) residue was reduced back to Met in two steps, firstly with 2 M $HBF_4$-thioanisole in TFA during the above acid treatment, and secondly with dithiothreitol during incubation of the unprotected peptide.

The crude peptides thus obtained were then successively purified by gel-filtration on Sephadex G-25 and semi-preparative HPLC. The two purified peptides exhibited single peaks on analytical HPLC. The two products possessed amino acids in ratios consistent with those predicted from the sequences of the two analogues after acid hydrolysis. The homogeneity of the peptides was checked by TLC, HPLC, amino acid analysis after 6 N HCl hydrolysis, and FAB-MS spectrometry. Physicochemical data for the synthetic analogues are shown in Tables 1 and 2.

**Table 1.** Characterization of synthetic thymosin $\beta_4$ analogues

| Peptide | Yield[a] (%) | $[\alpha]_D^{21}$ (c = 0.5, 1% AcOH) | TLC[b] Rf | FAB-MS[c] ($MH^+$) |
|---|---|---|---|---|
| [1-Nal$^{12}$]deacetyl-thymosin $\beta_4$ | 20 | −84.9° | 0.09 | 4971.13 |
| [Cha$^{12}$]deacetyl-thymosin $\beta_4$ | 23 | −79.5° | 0.11 | 4927.32 |

[a]Final yield after deblocking and purification starting from Fmoc-Ser(tBu)-Pam-resin.
[b]See the experimental section.
[c]Found values were in agreement with calculated values.

**Table 2**. Amino acid analysis of synthetic thymosin $\beta_4$ analogues[a]

| Peptide | Gly | Ala | Leu | Ile | Pro | Ser | Thr | Met[b] | Lys | Asp | Glu | 1-Nal | Cha |
|---|---|---|---|---|---|---|---|---|---|---|---|---|---|
| [1-Nal[12]]deacetyl-thymosin $\beta_4$ | 1.00 | 1.96 | 2.01 | 1.90 | 2.89 | 3.87 | 2.91 | 0.92 | 8.96 | 3.87 | 10.38 | 0.94 | |
| [Cha[12]]deacetyl-thymosin $\beta_4$ | 1.00 | 1.92 | 1.95 | 1.94 | 2.90 | 3.96 | 2.85 | 0.93 | 9.02 | 389 | 10.81 | | 0.96 |

[a]After acid hydrolysis with 6 N HCl at 126°C for 25 h.
[b]Met + Met(O).

**Table 3**. Effects of synthetic deacetyl-thymosin $\beta_4$ and its analogues on the impaired PHA stimulation of uraemic T-lymphocytes

| Peptide | No. of determinations | Dose (μg/ml) | SI[a,b] |
|---|---|---|---|
| —[c] | 3 | — | 274.2 ± 49.8 |
| —[d] | 3 | — | 114.3 ± 48.7[f] |
| Deacetyl-thymosin $\beta_4$[d,e] | 3 | 0.1 | 115.1 ± 49.3 |
| Deacetyl-thymosin $\beta_4$[d,e] | 3 | 1.0 | 198.3 ± 50.1[g] |
| [Phe(4F)[12]]deacetyl-thymosin $\beta_4$[d,e] | 3 | 0.1 | 190.6 ± 48.5[g] |
| [Phe(4F)[12]]deacetyl-thymosin $\beta_4$[d,e] | 3 | 1.0 | 230.5 ± 48.3[g] |
| [1-Nal[12]]deacetyl-thymosin $\beta_4$[d,e] | 3 | 0.1 | 179.7 ± 49.6[g] |
| [1-Nal[12]]deacetyl-thymosin $\beta_4$[d,e] | 3 | 1.0 | 216.8 ± 48.4[g] |
| [Cha[12]]deacetyl-thymosin $\beta_4$[d,e] | 3 | 1.0 | 117.9 ± 49.0 |
| [Cha[12]]deacetyl-thymosin $\beta_4$[d,e] | 3 | 10.0 | 115.3 ± 48.9 |

[a]Each value represents the mean ± SD of triplicate measurements.
[b]SI (stimulation index) was calculated according to the following formula: $SI = (I_2 - I_0)/(I_1 - I_0) \times 100$, where $I_2$ is mean fluorescence intensity of PHA-activated lymphocytes, $I_1$ is mean fluorescence intensity of PHA-nonactivated lymphocytes and $I_0$ is mean fluorescence intensity of ethidium bromide.
[c]Normal venous lymphocytes.
[d]Uraemic patients' lymphocytes.
[e]Incubation was carried out at 37°C in a humidified atmosphere of 5% $CO_2$ in air for 12 h in the presence of each peptide.
[f]$P < 0.03$, when compared with the normal subject using Student's t-test.
[g]$P < 0.01$, when compared with the uraemic patients using Student's t-test.

The immunological effects of the synthetic deacetyl-thymosin $\beta_4$, [Phe(4F)[12]]deacetyl-thymosin $\beta_4$, [1-Nal[12]]deacetyl-thymosin $\beta_4$ and [Cha[12]]deacetyl-thymosin $\beta_4$ were examined by the JIMRO (Japan Immunoresearch Laboratories Ltd) fluorometric blast-formation test. Responses of T-lymphocytes to mitogenic stimulation were significantly lower in uraemic patients that were those of normal persons. The *in vitro* effect of the synthetic peptides on the impaired PHA response of T-lymphocytes from uraemic patients is shown in Table 3.

### Discussion

Comparison of the stimulation index (SI) values of the blastogenic transformation of T-lymphocytes into lymphoblasts with mitotic activity upon PHA stimulation shows that in the case of the uraemic patients investigated, the synthetic analogue, [1-Nal[12]]deacetyl-thymosin $\beta_4$ which had a bulky naphthyl ring exhibited stronger restorative activity than that of our synthetic deacetyl-thymosin $\beta_4$, but it was a little bit weaker than that of [Phe(4F)[12]]deacetyl-thymosin $\beta_4$. However, the synthetic [Cha[12]]deacetyl-thymosin $\beta_4$ had no restorative effect even at a much higher concentration (Table 4).

Those results exhibited that not only 4-fluorophenyl ring of deacetyl-thymosin $\beta_4$ but also more bulky naphthyl ring of deacetyl-thymosin $\beta_4$ could bind with receptors of T-lymphocytes more strongly than a phenyl ring of deacetyl-thymosin $\beta_4$. On the contrary, another analogue, [Cha[12]]deacetyl-thymosin $\beta_4$ which contains an aliphatic ring at position of 12 instead of an aromatic ring showed no restorative effect. This result seems to suggest that aromaticity at position of 12 of thymosin $\beta_4$ plays significant roles for restorative activity on impaired blastogenic response of T-lymphocytes.

**Table 4**. Relative potencies of synthetic deacetyl-thymosin $\beta_4$ and its analogues on the impaired PHA stimulation of T-lymphocytes of uraemic patients

| Peptide | Relative potency (molar basis) |
|---|---|
| Deacetyl-thymosin $\beta_4$ | 1.00 |
| [Phe(4F)[12]]deacetyl-thymosin $\beta_4$ | 10.48 |
| [1-Nal[12]]deacetyl-thymosin $\beta_4$ | 9.86 |
| [Cha[12]]deacetyl-thymosin $\beta_4$ | — |

### References

1. Dobbelstein H. Immune system in uremia. *Nephron* 1979; **17**: 409–414.
2. Montgomerie JZ, Kalmanson GM, Guze LB. Renal failure and infection. *Medicine* 1968; **47**: 1–32.
3. Low TLK, Goldstein AL. Chemical characterization of thymosin $\beta_4$. *J Biol Chem* 1982; **257**: 1000–1006.
4. Dalakas MC. The role of thymosin $\beta_4$ and interdigitating cells in the thymic involvement of myasthenia gravis. *Ann NY Acad Sci USA* 1987; **84**: 809–812.
5. Abiko T, Sekino H. Deacetyl-thymosin $\beta_4$: synthesis and effect on the impaired peripheral T-cell subsites in patients with chronic renal failure. *Chem Pharm Bull* 1984; **32**: 4497–4505.
6. Abiko T, Sekino H. Synthesis of sex common amino acid sequence fragments of thymosins $\beta_4$, $\beta_8$ and $\beta_9$ and determination of their effects on the low E-rosette forming cells of lupus nephritis patients. *Chem Pharm Bull* 1984; **32**: 228–236.
7. Abiko T, Sekino H. Synthesis of calf thymosin $\beta_4$ fragment 16–38 and its effect on the impaired blastogenic response of a uremic patient with pneumonia. *Chem Pharm Bull* 1987; **35**: 3757–3765.
8. Abiko T, Sekino H. Synthesis of a new biological response modifier thymosin $\beta_4$ analogue and its restorative effect on depressed. *Mediators of Inflammation* 1992; **1**: 113–119.

**Received 28 October 1995;
accepted in revised form 29 November 1996**

## References

[PDF_00449] Abiko T., Sekino H. (1984). Deacetyl-thymosin beta 4: synthesis and effect on the impaired peripheral T-cell subsets in patients with chronic renal failure.. Chem Pharm Bull (Tokyo).

[PDF_00459] Abiko T., Sekino H. (1992). Synthesis of a new biological response modifier thyrnosin beta(4) analogue and its restorative effect on depressed.. Mediators Inflamm.

[PDF_00456] Abiko T., Sekino H. (1987). Synthesis of calf thymosin beta 4 fragment 16-38 and its effect on the impaired blastogenic response of T-lymphocytes of a uremic patient with pneumonia.. Chem Pharm Bull (Tokyo).

[PDF_00452] Abiko T., Sekino H. (1984). Synthesis of six common amino acid sequence fragments of thymosins beta 4, beta 8 and beta 9 and determination of their effects on the low E-rosette forming cells of lupus nephritis patients.. Chem Pharm Bull (Tokyo).

[PDF_00441] Dobbelstein H. (1976). Immune system to uremia.. Nephron.

[PDF_00444] Low T. L., Goldstein A. L. (1982). Chemical characterization of thymosin beta 4.. J Biol Chem.

[PDF_00442] Montgomerie J. Z., Kalmanson G. M., Guze L. B. (1968). Renal failure and infection.. Medicine (Baltimore).

